# Activating mechanosensitive channels embedded in droplet interface bilayers using membrane asymmetry†

**DOI:** 10.1039/d0sc03889j

**Published:** 2021-01-04

**Authors:** Robert Strutt, James W. Hindley, Jordan Gregg, Paula J. Booth, John D. Harling, Robert V. Law, Mark S. Friddin, Oscar Ces

**Affiliations:** Department of Chemistry, Imperial College London, Molecular Sciences Research Hub Shepherd's Bush London W12 0BZ UK o.ces@imperial.ac.uk; Institute of Chemical Biology, Imperial College London, Molecular Sciences Research Hub Shepherd's Bush London W12 0BZ UK; FabriCELL, Imperial College London, Molecular Sciences Research Hub Shepherd's Bush London W12 0BZ UK; Department of Chemistry, King's College London SE1 1DB London UK; Medicinal Chemistry, GSK Gunnels Wood Road, Stevenage SG1 2NY UK; Dyson School of Design Engineering, Imperial College London Imperial College Road SW7 2AZ UK m.friddin@imperial.ac.uk

## Abstract

Droplet microcompartments linked by lipid bilayers show great promise in the construction of synthetic minimal tissues. Central to controlling the flow of information in these systems are membrane proteins, which can gate in response to specific stimuli in order to control the molecular flux between membrane separated compartments. This has been demonstrated with droplet interface bilayers (DIBs) using several different membrane proteins combined with electrical, mechanical, and/or chemical activators. Here we report the activation of the bacterial mechanosensitive channel of large conductance (MscL) in a dioleoylphosphatidylcholine:dioleoylphosphatidylglycerol DIB by controlling membrane asymmetry. We show using electrical measurements that the incorporation of lysophosphatidylcholine (LPC) into one of the bilayer leaflets triggers MscL gating in a concentration-dependent manner, with partial and full activation observed at 10 and 15 mol% LPC respectively. Our findings could inspire the design of new minimal tissues where flux pathways are dynamically defined by lipid composition.

## Abstract 2

Electrophysiology shows asymmetric lysophosphatidylcholine-containing DIBs trigger mechanosensitive channel gating, enabling user-designed, autonomous flux pathways in droplet networks.

## Graphical abstract

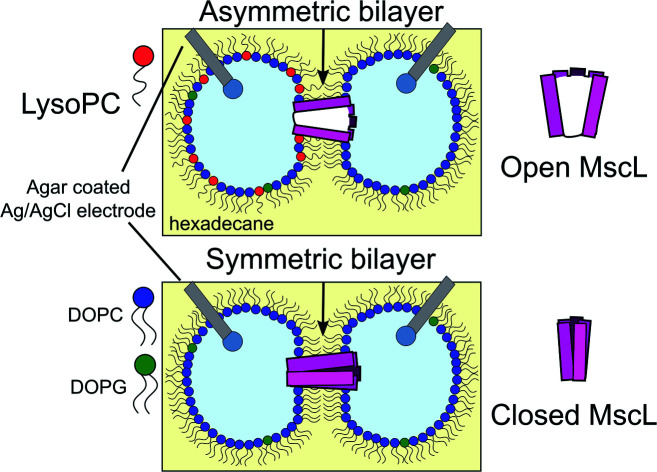

## Introduction

**Fig. 1:**
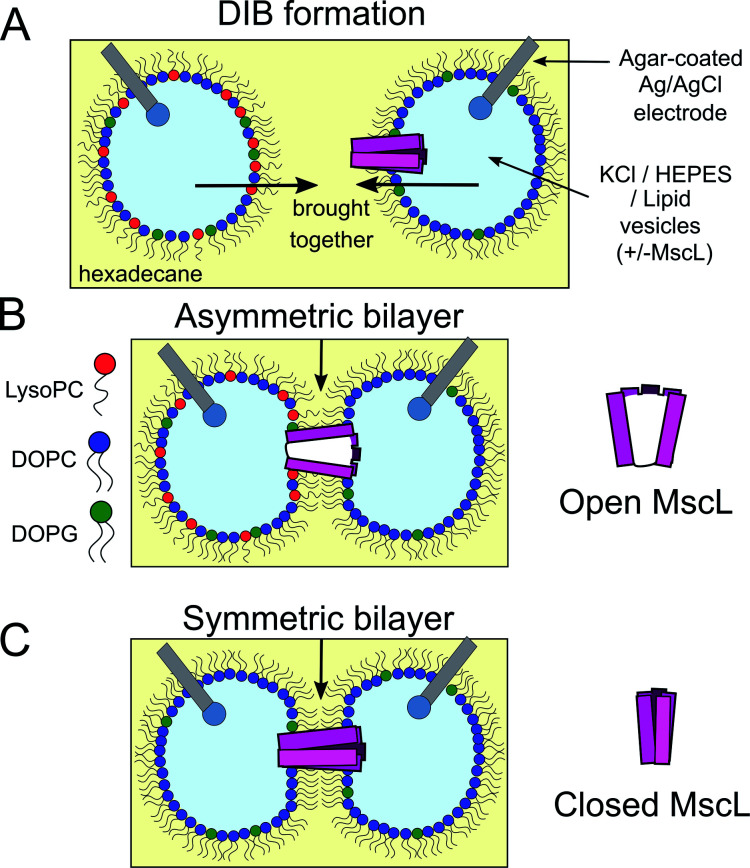
Probing MscL function in asymmetric droplet-interface bilayers (DIBs) using electrophysiology. (A) DIBs are formed by bringing together two lipid monolayer stabilised water-in-oil (w/o) droplets. As the two monolayers are brought into contact, a lipid bilayer spontaneously forms at the interface. Asymmetric bilayers can be easily assembled by supplying droplets with different lipids. (B) MscL can be incorporated by supplying vesicles containing reconstituted protein to the system, while inserting hydrogel-coated silver/silver chloride electrodes into each droplet facilitates electrical measurements of the membrane using electrophysiology. MscL is activated by the presence of LPC in one of the bilayer leaflets. (C) If symmetric bilayers are generated without LPC, reconstituted MscL channels remain shut, preventing flux of content across the DIB.

The field of bottom-up synthetic biology aims to reconstitute the form, function and behaviour of biological organisms from self-assembled chemical systems.^1–5^ To this end, different pathways have been explored to create compartmentalised biomimetic microstructures capable of supporting functions such as chemical synthesis,^6–8^ environment sensing,^9,10^ information transduction^11^ and motility.^12^ One route involves the use of lipid monolayer-stabilised water-in-oil (w/o) droplets, where contact between two droplets leads to the spontaneous self-assembly of a lipid bilayer at the interface (Fig. 1A). These structures, which are intended to mimic natural lipid membranes found in biology, are known as droplet interface bilayers^13,14^ (DIBs). DIBs offer several advantages over conventional planar bilayer systems (such as black lipid membranes (BLMs) or aperture suspended bilayers^15^), including increased stability,^14^ compartmentalisation of droplet content and the ability to support droplet volumes spanning three orders of magnitude from μl to pl.^16,17^

Unlike other approaches, the DIB platform also offers the option to assemble bilayer networks by connecting additional lipid-monolayer coated droplets in series, enabling the construction of minimal artificial tissues.^18–20^ By supplying different lipids to each droplet compartment,^21^ DIBs also offer a route for controlling membrane asymmetry, a feature that is ubiquitous in native biological membranes^22^ and is essential in facilitating core biological functions such as apoptosis and phagocytosis.^23^

This range of features combined with the ability to take quantitative measurements using either fluorescence microscopy or by electrophysiology (supported by hydrogel-coated silver/silver chloride electrodes inserted into each droplet) have led to the use of DIBs in a number of different studies concerning the properties of lipid membranes,^24–26^ the permeability of drug candidates/agrochemical compounds^27,28^ and the incorporation of membrane proteins.^29,30^ By using the DIB as an environment for the reconstitution of membrane proteins, the interplay between the protein and lipid bilayer can be interrogated *ex vivo* independently of the complex interaction networks found in cell biology. To-date multiple classes of membrane proteins have been reconstituted, including bacterial outer membrane proteins,^21^ ion channels^31^ and pore-forming peptide oligomers.^32,33^ In these systems, the inserted protein is typically activated by stimuli such as transmembrane potential,^34,35^ tension^36,37^ and/or pH,^38,39^ offering the user full control over the molecular flux across the membrane – a quality that can also be finely tuned through the use of mutants. Membrane asymmetry has also been shown to affect protein function, with an early study by Hwang *et al.* demonstrating the effect of charge asymmetry on the spontaneous gating probability of OmpG.^32^

The bacterial mechanosensitive channel of large conductance (MscL) is a membrane protein of interest due to its relatively large (∼3 nm, ∼3.5 nS) unselective pore size in the open-state. G22C F93W mutants enable the chemical activation^40^ and spectroscopic detection of MscL, whereas the V23T^41^ and G22S^42^ gain-of-function (GOF) mutants offer lower tension thresholds for activation (∼6 mN m^−1^). Although both GOF mutants have been reconstituted and activated in DIBs^41–45^ with greater ease than their wild-type counterpart, they still require individual droplets to be prepared or manipulated by the user, meaning that flux pathways cannot be defined in real-time. This becomes a problem when constructing droplet networks, especially given that network architecture and composition can be used to define the flow of molecular information throughout the tissue.^46^ To this end, recent work has focused on using external stimuli such as light^19^ or temperature^47^ to dynamically control network activation, however this has only been achieved for the water-soluble alpha-toxin alpha haemolysin and not for water-insoluble membrane proteins, highlighting the need to develop methods that offer new, orthogonal ways to define information flow across a bilayer network.

Here we show for the first time in a droplet system that the activation of MscL can be achieved using bilayer asymmetry, *i.e.* in the absence of any channel activators or applied external pressure. We achieve this by incorporating 1-oleoyl-2-hydroxy-*sn*-glycero-3-phosphocholine (LysoPC/LPC) into one of the bilayer leaflets (Fig. 1B and C) to generate an asymmetric change in the lateral pressure profile that has been shown previously to activate the MscL channel,^48–53^ and identify protein activity using single-channel electrical measurements (Fig. S1†). Our method to control the full gating of the G22C F93W loss-of-function (LOF) channel in a DIB system using membrane patterning could be applied to other mutants and serve as a new strategy to control molecular flux in droplet networks, helping to design and build minimal tissues capable of increasingly complex information processing.

## Results

### Activity of MscL G22C F93W in lipid vesicles

MscL G22C F93W was expressed in BL21(DE3) *E. coli* and purified *via* cobalt-immobilized affinity chromatography as in previous work^10,50^ (Fig. S2†). The activity of the expressed MscL channel was tested through reconstitution into 1 : 1 (mol : mol) 1,2-dioleoyl-*sn*-glycero-3-phosphocholine (DOPC) : 1,2-dioleoyl-*sn*-glycero-3-phospho-(1′-*rac*-glycerol) (DOPG) lipid vesicles containing a self-quenching (50 mM) concentration of entrapped calcein.^54^ MscL-vesicles were produced *via* thin-film hydration, extrusion and detergent mediated reconstitution, before separating unencapsulated calcein from the produced vesicles *via* size-exclusion chromatography to form vesicles ∼100 nm in diameter (Fig. S3†).

**Fig. 2:**
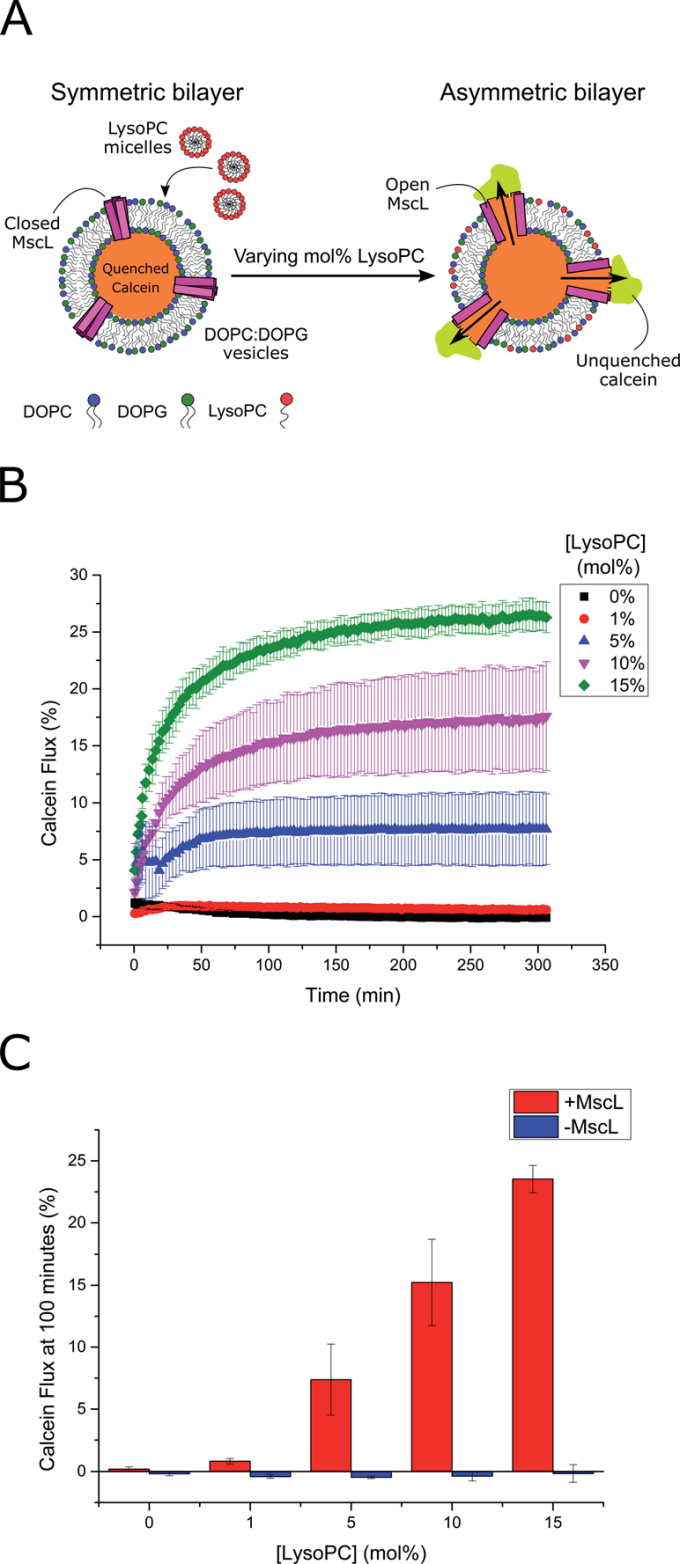
LPC triggers MscL activation in lipid vesicles. (A) MscL is reconstituted into calcein-loaded lipid vesicles *via* detergent-mediated reconstitution. By encapsulating calcein at high concentrations, its fluorescence is fully quenched. Asymmetric insertion of LPC into the outer leaflet of the vesicle membrane is facilitated by the addition of LPC micelles to solution. If MscL is activated by LPC insertion, calcein will diffuse through the channel out of the vesicle where it will become diluted and generate a fluorescent signal as it becomes unquenched. (B) Calcein flux (%) over 5 hours from MscL-vesicles upon the addition of increasing mol% LPC to solution. LPC-dependent calcein flux can be observed indicating successful MscL activation by LPC. (C) MscL is critical for calcein flux. If calcein flux for vesicles ± MscL is assessed 100 minutes after LPC addition, negligible flux is observed at all LPC concentrations for vesicles lacking the channel. Error bars for (B) and (C) represent 1 SD (*n* = 3).

MscL channel activity was then assayed *via* three different activation methods in parallel: (i) chemical modification of vesicle structure through the addition of LPC (Fig. 2A) (ii) chemical modification of vesicle structure through the enzymatic activity of secretory phospholipase A2 (sPLA2)^55^ and (iii) chemical modification of the MscL channel through addition of the channel activator [2-(trimethylammonium)ethyl]methanethiosulfonate (MTSET). An LPC concentration gradient was added to vesicles ± MscL, and the calcein flux response was monitored over 5 hours (Fig. 2B). Vesicles containing the MscL channel displayed calcein flux that increased as a function of increasing LPC concentration, indicating that the lysolipid could successfully activate the channel. When the same LPC concentrations were added to vesicles lacking the MscL channel, negligible calcein flux occurred over the 300 minute experimental lifetime (Fig. 2C and S4†), indicating that the channel is essential for triggered release in response to LPC, and that the expressed MscL protein is active.

Based on these flux measurements, the threshold concentration of LPC necessary for channel activation lies between 1–5 mol% LPC. This value agrees well with previous investigations of the effect of LPC on MscL reconstituted into vesicles,^49,51,53^ where less than 10 mol% LPC was necessary for channel activation.^53^ To further confirm channel activity, enzymatic and chemical activation of the reconstituted channel was undertaken using sPLA2 and MTSET respectively (Fig. S5†). Again, the presence of the MscL channel enabled calcein flux responses to enzymatic and chemical (channel labelling) stimuli, whilst negligible release was observed for vesicles lacking the channel, indicating high activity of the channel.

### LPC can activate MscL channels reconstituted into DIBs *via* membrane asymmetry

After confirming the activity of expressed MscL, the effect of LPC on MscL-functionalized DIBs was studied. MscL was reconstituted into vesicles with lower charge better suited for DIB formation (95 : 5 DOPC : DOPG vesicle composition), and used as the droplet-stabilising lipid source (lipid-in) for w/o droplets (1 μl). Droplets were manipulated manually on the tips of agar-coated silver/silver chloride electrodes to assemble DIBs and the membrane was probed using droplet electrophysiology (Fig. 1A and S1†) exactly as described in our previous work, applying a −100 mV potential across the bilayer.^56^

**Fig. 3:**
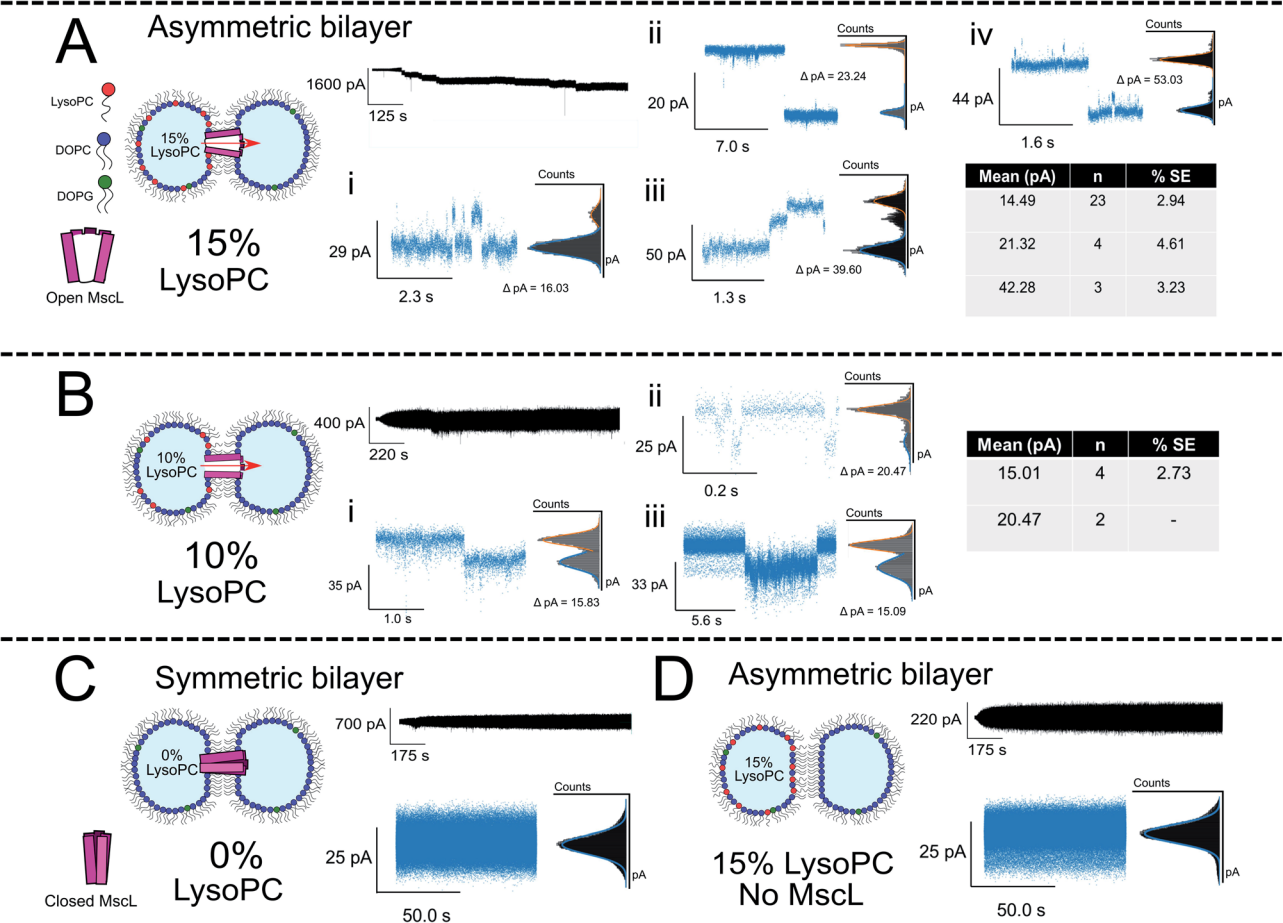
Membrane asymmetry gates MscL G22C F93W channels reconstituted into DIBs. (A) Asymmetric MscL DIBs containing 15 mol% LPC are formed by incubating LPC-containing vesicles in the left droplet of the DIB (PC : PG : LPC 80 : 5 : 15) and MscL-containing vesicles in the right droplet (PC : PG : LPC 95 : 5 : 0). Each 1 μl droplet additionally contained 20 mM HEPES, 100 mM KCl pH 7.4 buffer. The activation of sub conducting states was observed, clustered into three main gating events ∼14, 21 and 42 pA that are attributed to sub-conducting states of the MscL channel (*n* = 4). Further events for 15 mol% LPC discussed in Fig. 4. (B) Reducing the LPC concentration in the asymmetric DIB attenuates channel function. An ∼10-fold drop in gating events are observed when LPC is reduced to 10 mol%, and no fully open states are recorded for the MscL channel (*n* = 3). (C) If a symmetric MscL DIB is generated without LPC, no gating events are observed indicating that the MscL channel is closed (*n* = 2). (D) If asymmetric LPC DIBs are prepared without MscL, no gating events are observed, indicating that in (A) flux across the bilayer is controlled by the response of MscL to membrane asymmetry (*n* = 3). Cartoons illustrate lipid and protein compositions. Black traces show examples of full, 50 kHz recorded trace for each composition with featured zoom in to indicate channel activity. Zoom in data shown with an applied low pass filter at 2.5 kHz. All traces recorded at −100 mV. The percentage standard error of the mean is denoted by % SE.

To generate asymmetric DIBs, the first droplet was formed containing PC : PG : LPC vesicles with increasing LPC concentrations (5, 10 and 15 mol%), whilst a second droplet was formed containing PC:PG vesicles ± MscL (Fig. 3A, B and S9† for 15, 10 and 5 mol% LPC traces respectively). Asymmetric DIBs incorporating MscL showed LPC-dependent gating behaviour, with 10-fold greater gating events occurring in MscL DIBs containing 15 mol% *vs.* 10 mol% respectively. We observed negligible gating events in DIBs containing 5 mol% LPC (#events = 42/6/1 for 15/10/5% LPC respectively). These events could be clustered into seven gating states, where each state is defined as a function of the percentage of full channel conductance (*S*%). Six of these states align with previously proposed conductance states for the channel^57,58^ (Table S1 and Fig. S6†). A histogram analysis approach was used to characterise the step changes of all traces (see Note S1 for further information†).

The majority of events in our 15% LPC results occurred at S4.5 ∼14 pA (0.14 nS, *n* = 23) and S6.6 ∼21 pA (0.21 nS, *n* = 4). We additionally observed events occurring at S13 ∼42 pA (0.42 nS, *n* = 3), which we assign to the sub-conducting state of MscL observed in our previous work.^56^ At times, we saw combinations of such states leading to the observation of events ∼55 pA (Fig. 3A(iv)), which could represent the gating of two separate MscL proteins or a change in the conformational state of a single channel. Statistical testing between these three states observed in 15% LPC DIBs confirms that the events can be clustered into three populations in this manner (*p* < 0.001), as well as confirming that the S4.5 sub-conductance state was observed in both 10 and 15% LPC DIBs respectively (*p* < 0.001). As only the lowest two sub-conductance states are observed at 10% LPC, we conclude that 10% LPC is able to only partially gate the channel.

As mentioned above, the observed gating events ∼42 pA correlate well with our previous work^56^ which is established as S12, a subconductance state ∼12% open conductance.^58^ Similarly, the low sub-conductance state of S6.6 has been previously observed in MscL gating studies.^57^ We note that subconductance states were maintained for extended lengths in our recorded 15% LPC traces (Fig. S7B and C†), indicating that the combination of MscL and asymmetric LPC may be useful for sustained flux across a DIB. Interestingly, the most frequent subconductance state observed here (S4.5) has not been observed in previous MscL electrophysiology experiments. Its appearance here is likely due to the combination of using a loss-of-function channel mutant under high potential (−100 mV), which has been shown to increase sampling of low conductance states.^57^

**Fig. 4:**
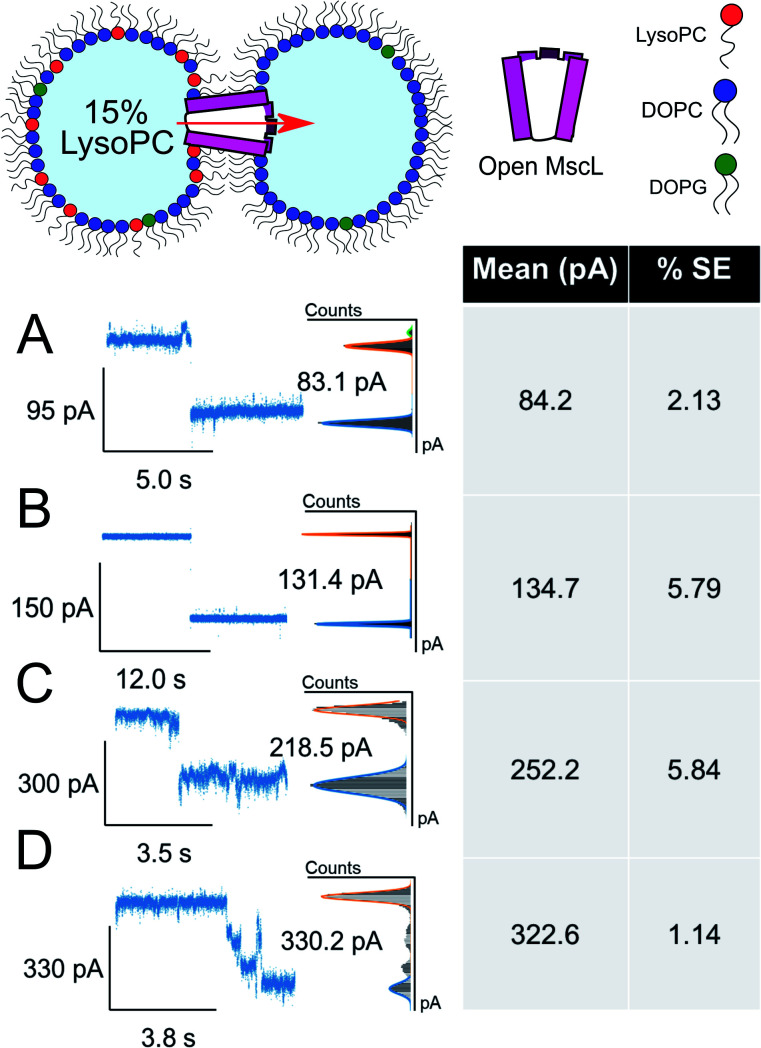
Large ΔpA MscL gating events are observed in the presence of 15 mol% LysoPC. Divided into 4 clusters, large gating events are also observed in asymmetric DIBs formed with 15 mol% LysoPC and MscL. All data show example traces with mean and SE of grouped gating events presented (*n* = 3) (A–C). Representative gating events at 84, 135 and 252 pA are observed. (D) Full opening of MscL at 323 pA passing through distinct sub states as indicated by sustained regions in the total pA change. Cartoons illustrate lipid and protein compositions. The percentage standard error of the mean is denoted by % SE.

The asymmetric incorporation of 15% LPC in MscL DIBs also led to the occupation of higher gating states (Fig. 4). The states shown in Fig. 4A–C are attributed to the higher subconductance states of the channel, showing good agreement with previously established conductances for these states^58^ (Table S1 and Fig. S6†). The open state of the channel (S100 ∼3.2 nS, *n* = 3) was observed to occur through these larger subconductance states (Fig. 4D, S13 and S14†), indicative that asymmetry can drive full opening of the MscL channel. We note that LPC flip-flop has previously been shown to be negligible on similar timescales to those within our system.^59,60^ Here, LPC flip-flop appears insignificant as MscL gating occurs throughout the ∼30 min experimental timescale (Fig. 3A).

If DIBs were produced containing MscL but without LPC, negligible gating events occurred, confirming that the protein is in the closed state in the absence of LPC (Fig. 3C). This agrees with our previous work, where gating of reconstituted MscL in symmetric DIBs was not observed.^56^ Similarly, if DIBs were produced without MscL but LPC asymmetry was maintained (Fig. 3D), no gating events were detected, indicating that gating could not be attributed to transient pore formation in the DIB caused by the presence of non-bilayer forming LPC.^61^

To probe the role of LPC asymmetry further, control experiments with symmetric DIBs containing 15 mol% LPC were performed. This composition attenuated bilayer formation resulting in poor DIB success rates (*n* = 1/6). When formed, the recordings indicated bilayer instability such that protein activity could not be delineated from the trace (Fig. S12†). All unfiltered traces for our complete data set can be observed in Fig. S7–S12† in the ESI.†

The increased likelihood for occupying sub-conductance states was not observed in previous work where patch-clamp electrophysiology was used to analyse the effect of LPC on MscL.^51^ We attribute this to differences in experimental setup between excised bilayer patches in patch-clamp and the DIB membrane utilised in droplet electrophysiology, the high applied potential of −100 mV leading to increased occupancy of subconductance states^57^ and the LOF mutant used in the study (WT *vs.* G22C F93W here). Whilst further optimisation is necessary to generate more digital activation behaviour, our electrophysiology experiments indicate that LPC can be successfully employed to gate MscL reconstituted into asymmetric DIB membranes.

## Discussion

We have presented the first evidence that bilayer asymmetry alone can be used to gate MscL channels reconstituted into DIBs. Two aspects of our electrophysiology results indicate that LPC-gating of MscL could represent a powerful tool in controlling flux through DIB networks. Firstly, we see extended gating of the MscL channel in LPC bilayers on the minute timescale (Fig. 3A, S6B and C, S13 and S14†) which is ideal for sustained flux across the membrane. This is activated without externally applied tension, and hence could be used without the presence of any external mechanical actuation. The lower sub-conductance states observed here correlate with our previous electrophysiology work on the same mutant (triggered *via* MTSET-labelling of the channel)^56^ and this has been shown to be sufficient for the flux of calcein (MW = 622.6 Da) across MscL DIBs^62^ and gating of solutes ∼6.6 kDa through MscL reconstituted into lipid vesicles.^63^ We can therefore infer that LPC-gated MscL DIBs should enable molecular flux with a MWCO between 0.6 and 6.6 kDa (and potentially higher than this based on our higher conductance gating events which generate the ∼3 nm diameter of the open MscL pore^64^).

Secondly, the flux we have observed is actuated purely by asymmetry in the lipid bilayer and could be patterned during network assembly, or tuned dynamically using optical tweezers as demonstrated recently using vesicles.^65,66^ Furthermore, LPC flip-flop could be used to build pre-defined flux patterns into the network, as flip-flop to a symmetric bilayer should close the MscL channel.^48^ The slow kinetics of this process^67^ could be potentially altered if combined with LPC-chelators such as BSA that have been shown to deactivate MscL channels upon removal of LPC from the membrane.^53^

The activation mechanism of MscL in response to asymmetric membrane incorporation of LPC has been previously indicated to occur *via* asymmetric changes in the lateral pressure profile of the membrane, and not via-stretch-induced tension in the membrane.^48,51^ In our DIB setup, the global curvature of the membrane is insignificant from the perspective of a single channel, whilst all our experiments are conducted without applied pressure or mechanical stimulation. We therefore attribute the gating observed here to an asymmetric curvature stress induced by LPC in the DIB perturbing the lateral pressure profile at the water–lipid interface. Such a change in membrane lateral pressure reduces the gating energy for the channel, enabling spontaneous gating without applied pressure.^68^

MD simulations have indicated that the asymmetric presence of LPC can generate areas of high local curvature in the membrane that contribute to channel activation.^69^ This would be energetically less likely to occur in DIBs compared to vesicles, as DIBs possess ∼30–60-fold higher surface tension (∼1–2 mN m^−1^ (ref. ^26^) *vs.* ∼30 μN m^−1^ (ref. ^70^)). Such an effect may decrease the probability of channel activation in DIBs compared to vesicles assuming local curvatures are truly necessary. Comparative electrophysiology experiments on the same MscL channel in both asymmetric DIBs and patch-clamp setups may help further elucidate the mechanism of LPC-activation of MscL.

To gain further insight to our system the monolayer surface tension of aqueous droplets stabilised with PC:PG:LPC vesicles containing 0–15 mol% LPC was quantified using droplet shape analysis (Fig. S15†). A linear decrease in surface tension was observed from 0–15 mol%, with the surface tension difference between a droplet containing 0 and 15% LPC found to decrease by 0.74 mN m^−1^, from 1.83 ± 0.23 mN m^−1^ for 0 mol% LPC to 1.09 ± 0.71 mN m^−1^ for 15 mol% LPC. This tension decrease is expected considering that LPC acts as a surfactant, and measured tensions are similar to those of previously studied asymmetric DIBs (1–2 mN m^−1^ (ref. ^26^)). Indeed, a similar decrease in tension in response to LPC has been shown previously for phosphatidic acid-stabilised droplets.^71^ These results appear to match well with the predicted effect of asymmetric LPC generating a pressure differential across the leaflets of a DOPC membrane.^69^ This further indicates that the primary driver of channel activation observed here is leaflet asymmetry and not simply a high bilayer surface tension, as DIB surface tensions are an order of magnitude lower than the activation tension of the channel (>12–14 mN m^−1^ (ref. ^51^)).

We note that the threshold activation concentration of LPC required for MscL gating differs between the vesicle and DIB model systems: gating occurs from 5 mol% LPC added to MscL vesicles, whilst 10 mol% LPC is necessary when the channel is reconstituted into DIBs. We hypothesise that the increased activation threshold may be due to LPC partitioning between the DIB and droplet monolayers or into the bulk hexadecane.^72^ These mechanisms would reduce the effective LPC concentration in the bilayer to minimise the destabilising effect of the lyso-lipid^73^ and hence minimise the free energy of the system.

Although we see full gating of the MscL channel in our work, the most frequently observed events appeared to be gating *via* sub-conductance states. This could be due to LPC partitioning, higher applied voltage or may reflect the high activation energy of the mutant used here.^51^ The sidedness of MscL insertion may also play a role: MscL is reconstituted without preference into vesicles (and hence into the DIB), and the presence of both orientations in the same membrane may affect which channels are gated by LPC asymmetry as well as channel gating probability. Interestingly, the amphipath 2,2,2-trifluoroethanol was recently shown to activate MscL when added to either leaflet of a patch-clamp setup,^74^ indicating that LPC may also be able to activate both channel orientations in the DIB. Further investigation could be conducted by using protocols which reconstitute MscL in a single orientation into the DIB membrane as well as testing the LPC activation mechanism with wild-type or GOF channel mutants with lower gating energies.^51^ Indeed, MscL V23T has shown a higher probability for the open state when reconstituted in DIBs in response to mechanical actuation,^44^ and channel activation should be feasible by using LPC to create asymmetric bilayers.

Recently, MscL V23T was shown to possess voltage-dependent gating in asymmetric DPhPC:DOPhPC DIBs under mechanical stimulation when negatively hyperpolarized.^45^ This was achievable for the V23T mutant due to a dominant dielectric effect compared to the WT channel on account of increased pore solvation. The G22C F93W channel possesses increased pore hydrophobicity (and hence decreased pore solvation^75^) compared to the WT, reducing the dielectric component of the channel in the presence of an electric field compared to both V23T and WT. Charge asymmetry is therefore significantly unlikely to gate either the WT or G22C MscL channels without applied tension, but such asymmetries may affect the onset of MscL gating to an LPC asymmetry. This may be useful as a tool to further differentiate channel function in droplet networks.

## Conclusions

In conclusion, we have shown that LPC-induced asymmetry in DIB membranes can be utilised as a tool for the activation of incorporated mechanosensitive channels in a sustained manner. This work furthers recent application of the MscL protein as a model mechanosensitive channel ‘part’ for use in bottom-up synthetic biology,^10,76,77^ extending its utility in droplet-interface bilayer networks. The combination of responsive protein channels and bilayer asymmetry shown here points a way towards using lipid composition to define network function – a still underexplored parameter in the design of droplet systems and minimal tissues.

## Conflicts of interest

There are no conflicts to declare.

## Supplementary Material

SC-012-D0SC03889J-s001
