# Supplementary material for: Activating mechanosensitive channels embedded in droplet interface bilayers using membrane asymmetry
Source: Chem Sci. 2021 Jan 4;12(6):2138–45. doi: 10.1039/d0sc03889j (PMC8179348; doi:10.1039/d0sc03889j)
Supplement: SC-012-D0SC03889J-s001 [file SC-012-D0SC03889J-s001.pdf]

Electronic Supplementary Material (ESI) for Chemical Science.
This journal is © The Royal Society of Chemistry 2020

Electronic Supporting Information for:

Activating mechanosensitive channels embedded in droplet interface bilayers using membrane asymmetry

Robert Strutt [a],[b]†, James W. Hindley[a],[b],[c]†, Jordan Gregg[a], Paula J. Booth[c],[d], John D. Harling[e], Robert V. Law[a],[b],[c], Mark S. Friddin[a],[f]* and Oscar Ces[a],[b],[c]*

Materials and Methods

1,2-dioleoyl-sn-glycero-3-phophocholine (DOPC), 1,2-dioleoyl-sn-glycero-3- phosphoglycerol (DOPG) and 1-oleoyl-2-hydroxy-sn-glycero-3-phosphocholine (LPC) were obtained from Avanti Polar Lipids™ (USA). Detergent Octyl-β-D-Glucopyranoside (OG) was purchased from Affymetrix (USA). SM-2 Adsorbant BioBeads were purchased from BioRad (USA). Phospholipase A_2 (sPLA_2) from honey bee venom (*Apis mellifera*) (#P9279) was obtained from Sigma-Aldrich (UK). (2-(Trimethylammonium)ethyl methanethiosulfonate, bromide (MTSET) was purchased from Biotium (USA). All other reagents were purchased from Sigma Aldrich (UK).

Expression and purification of recombinant MscL G22C F93W

MscL was expressed as previously described[1]. Briefly, *E. coli* BL21 (DE3) cells carrying the pET28a (Novagen) vector with chemically gated MscL gene (G22C and F93W mutations) were grown overnight in Luria broth (LB) (30μg/ml kanamycin) at 37°C and 250 rpm. The overnight culture was then reseeded 1:100 into 1L fresh LB (30μg/ml kanamycin) before growing at 37°C and 250 rpm to the maximum of the exponential phase (OD_{600} ~ 1).

Protein production was then induced for 90 min in the presence of isopropyl β-D-thiogalactopyranoside (0.5 mM) before purifying the resultant MscL via high pressure lysis (two passages at 25 kpsi), isolation of the membrane fraction through centrifugation (100,000g, 1hr, 4°C), and solubilisation of the membrane fraction overnight at 4°C using 2% (w/v) DDM in 20 mM HEPES, 100 mM KCl pH 7.2 buffer. Protease inhibitors were used throughout the process to minimise degradation of expressed protein. The next day, insoluble material was removed by centrifugation (100,000g, 45 min, 4°C) before binding the solubilised fraction to TALON cobalt metal affinity resin (4 ml of 50 wt% resin). A low concentration of imidazole (6 mM) was then used to elute non-specifically bound proteins before increasing the concentration (150 mM) to elute the expressed MscL. Imidazole was then removed via buffer exchange on a PD-10 desalting column (GE Healthcare) before centrifugal concentration of the protein to obtain purified MscL pentamer at a final concentration of ~0.15 – 1.5 mM MscL pentamer (as ascertained via tryptophan absorbance at 280 nm enabled by the F93W point mutation). The protein was flash-frozen and stored at -80°C until use.

Preparation of PC:PG lipid vesicles containing reconstituted MscL

DOPC:DOPG at a molar ratio of 50:50 and 95:5 were identically prepared. Films (5 mg, ~ 6 μmol total lipid) were weighed out and dissolved in chloroform. This lipid solution was then gently mixed for 2 min before evaporating the chloroform under a stream of N2(g) and storing the resultant film under vacuum overnight at room temperature. Films were rehydrated with 40 mM octyl-β-D-glucopyranoside (OG), 20 mM HEPES, 100 mM KCl at pH 7.4 to a concentration of 10 mg/ml and freeze-thawed 5 times. If preparing vesicles for calcein experiments, the buffer also contained calcein at a concentration of 50 mM.

The produced multilamellar vesicles were extruded through 0.1 μm polycarbonate filters 21 times to produce a suspension of large unilamellar vesicles ~ 100 nm in diameter (Figure S3). Vesicles were then added to MscL at a 50,000:1 lipid:protein molar ratio, and left to mix at 45 minutes at 4°C. OG was then removed through the addition of 300 mg of SM-2 Bio-Beads (mesh size 25-50, Bio Rad (USA)) in 3 x 100 mg batches, leaving the lipid-MscL-OG suspension on rotator bars for 1 hour/batch of beads at 4°C. Vesicles were purified by size-exclusion chromatography using a Sephadex G-50 column, eluting the sample with sucrose buffer (500 mM sucrose, 100 mM KCl, 20 mM HEPES, pH 7.4) in fractions of 300μl. Control samples (-MscL) were prepared identically except with the addition of 0.13 % DDM.

Spectroscopic Activity Testing of MscL

The fluorescence of all vesicle samples was recorded in 96-well plates, with calcein fluorescence emission recorded at $\lambda_{ex/em}$ = 494/514 nm. Vesicles ± MscL were diluted in sucrose buffer at a 1:50 (v/v) ratio. Background measurements (F_0) were collected prior to reagent addition in all experiments to confirm the stability of produced vesicles. MscL activity was then tested through the addition (in parallel) of LPC (added to a final concentration of 0-15 mol% (of ~125 μM lipid content present in each well)), $sPLA_2$ (0.5 nM final concentration) or MTSET (1 mM final concentration). Equivalent volumes of buffer was used as negative controls in all cases, and fluorescence was monitored in each case for 300 minutes.

Triton X-100 (3 v/v%) was added at the end of each assay, left for 15 minutes to enable complete vesicle lysis and then the samples were imaged for a further 10 minutes (complete lysis was established by negligible change in fluorescence during final imaging). Measurement of lysed vesicles provides the maximum fluorescence value for each well (F_{END}), allowing for normalization of results. Since the amount of vesicles in each well remains constant, the percentage and rate of fluorescence increase can be directly related to the release of dye through open MscL pores[2,3]. Normalized fluorescence data was obtained using equation (1), where F_t is the fluorescence value at time t:

$$Calcein\ Flux\ (\%) = \frac{F_t - F_0}{F_{END} - F_0} * 100 \qquad (\mathbf{1})$$

Platform for DIB formation

Following from our previous work[4], our approach to collecting electrical measurements across a DIB is as follows - gaskets with 10 mm through holes were laser cut from 5 mm thick poly-methyl-methacrylate (PMMA) (Weatherall, Wendover, UK) prepared with double sided adhesive and stuck to polydimethylsiloxane (PDMS) coated glass slides. These were filled with hexadecane to act as a well for DIB formation. Ag/AgCl electrodes were coated with 5% agar (w/v) in ultrapure water, washed with HEPES/KCl buffer before pipetting 1 μl of proteoliposome or liposome solution onto the electrodes. The electrode which supported proteoliposome solution was kept consistent across experiments. Droplets were lowered into the hexadecane well within the gasket and incubated for 5 – 10 mins before being manipulated into contact to encourage DIB formation. Extensive washing with buffer of the electrodes was performed between experiments.

Electrophysiology

Electrodes were connected via a head stage to an Axopatch 200B amplifier and a Digidata 1440A digitsizer. A holding potential of – 100 mV with a 50 kHz sampling rate and a 5 kHz low pass Bessel filter were used to obtain current measurements across the DIB. Data was then analysed simultaneously in ClampFit (Molecular Devices) or utilising the pyabf library in python (See note S1 for details on python analysis).

Pendant drop measurements and drop shape analysis (DSA)

Following from previous work within our group, droplet shape analysis (DSA) experiments were conducted using a Krüss EasyDrop pendant tensiometer.[5] Lipid in droplet compositions, prepared as described prior, were loaded into a 1ml syringe equipped with 0.52 mm flat needle. The needle was lowered into a cuvette filled with hexadecane and droplets were pipetted out. Droplet volumes were kept low, in line with previous work.[5] An aqueous density of 1000 kg m^{-3}, hexadecane density of 770 kg m^{-3} and an acceleration of gravity of 9.8 m s^{-2} were employed. As has been shown suitable prior,[6] these droplets were incubated for ~8 minutes to allow monolayer equilibration, before images were captured and analysed with OpenDrop software to determine the interfacial tension (IFT – mN/m), droplet volume (mm^3) and Worthington number (Wo).[7]

Supplementary Data

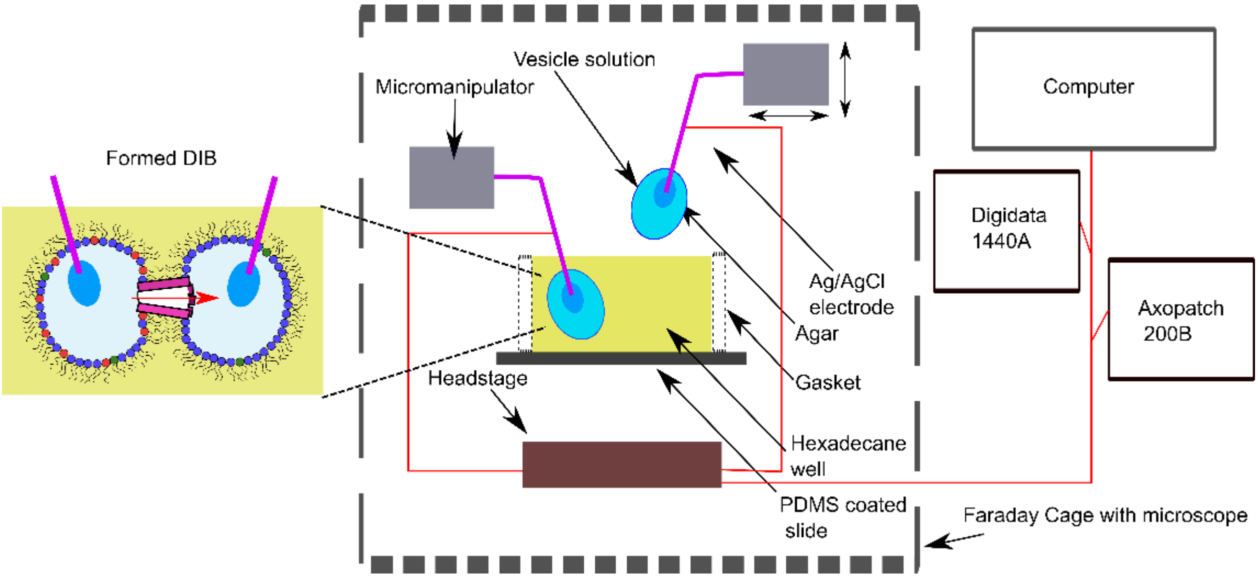

Figure S1. Electrophysiology setup. Cartoon depicting the set up to form a DIB. Digitizer (Digidata 1440A) and Amplifier (Axopatch 200B) attached via a shielded wire to a headstage, attached via gold plated wire to Ag/AgCl electrodes. Within a faraday cage the system is mounted on the base of a microscope and the electrodes are mounted on micromanipulators to control droplet orientation in 3D. Once vesicle solution is pipetted onto agar, the droplets are lowered into a hexadecane well where they are incubated to form a monolayer and brought together to form a DIB where asymmetric vesicle composition dictates asymmetric DIB formation.

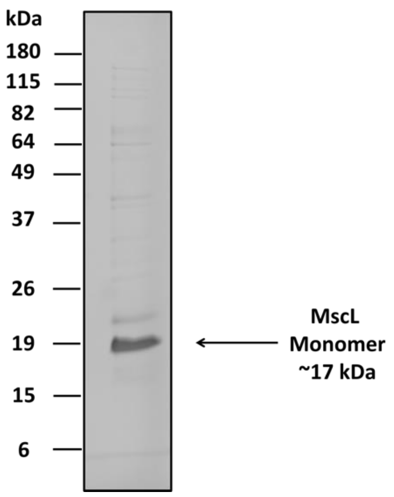

Figure S2. Denaturing sodium dodecylsulphate - polyacrylamide gel electrophoresis of recombinant MscL G22C F93W. Purification via Co^{2+} immobilised metal affinity chromatography yields high concentrations of MscL protein ~ 17 kDa in weight. Bands at higher molecular weight are attributed to MscL oligomers present due to the high concentration of loaded MscL, whilst the band above MscL could represent trace contamination by SlyD[10]. This should not impact use of MscL in this work as: i) MscL is diluted 200-fold from the concentration assayed here when reconstituted into lipid vesicles, and ii) vesicle samples are purified via size-exclusion chromatography before use, separating vesicles from all unencapsulated molecules in solution up to the 30 kDa exclusion limit of Sephadex G-50.

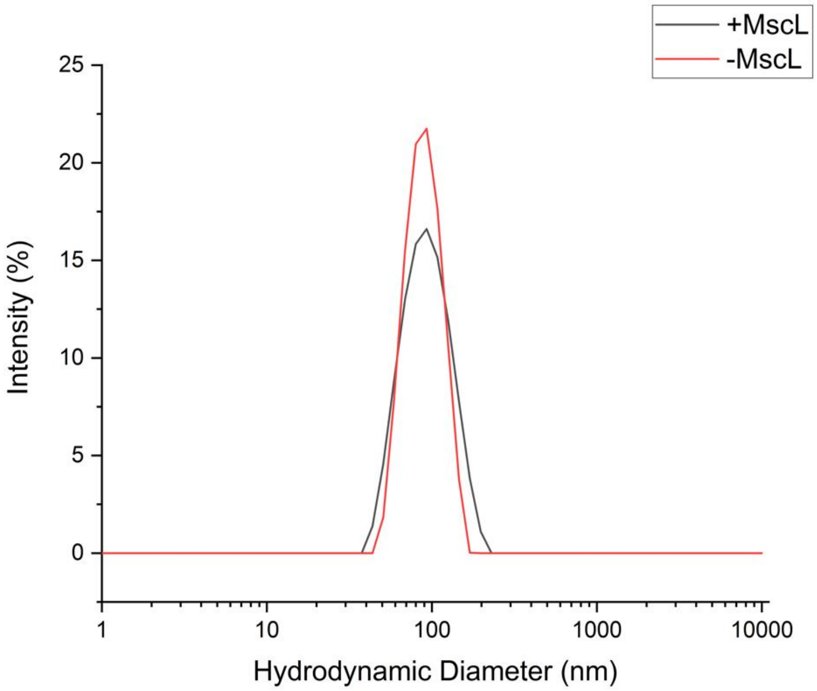

Figure S3. Dynamic Light Scattering of 1:1 DOPC:DOPG vesicles +/- MscL. Representative DLS traces from an MscL reconstitution. Reconstitution of the MscL channel does not significantly affect the size or polydispersity of formed vesicles. +MscL: size-average = 96.5 nm, polydispersity index = 0.121. -MscL: size-average = 91.2 nm, polydispersity index = 0.0844.

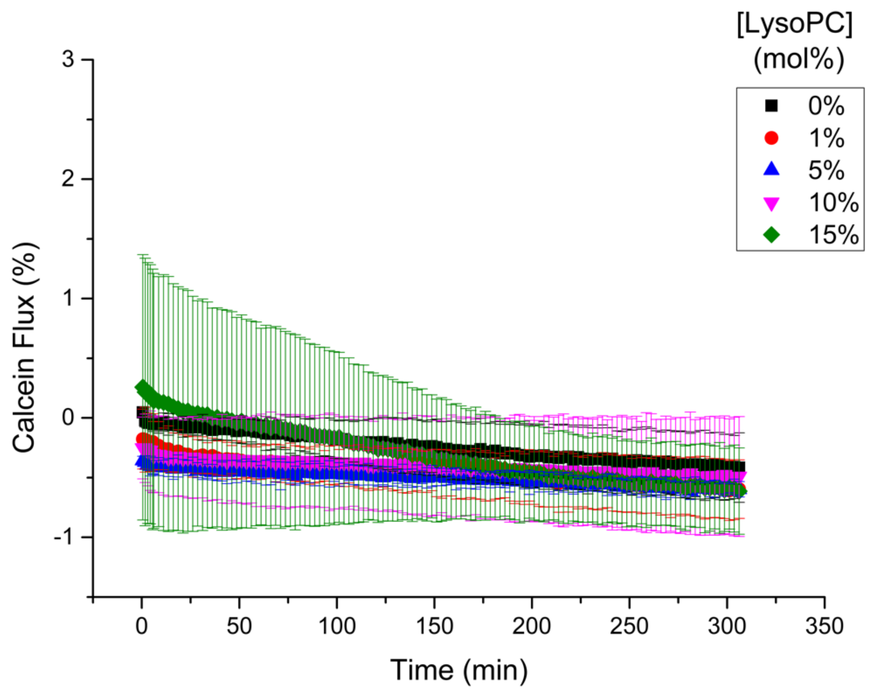

Figure S4. Effect of lysoPC micelle gradient on DOPC:DOPG vesicles lacking MscL. For vesicles prepared without MscL (equivalent volume of 0.13(w/v)% dodecylmaltoside micelles added during detergent-mediated reconstitution), negligible calcein flux is observed at all lysoPC concentrations added to purified vesicles. Error bars = 1 S,D., n =3.

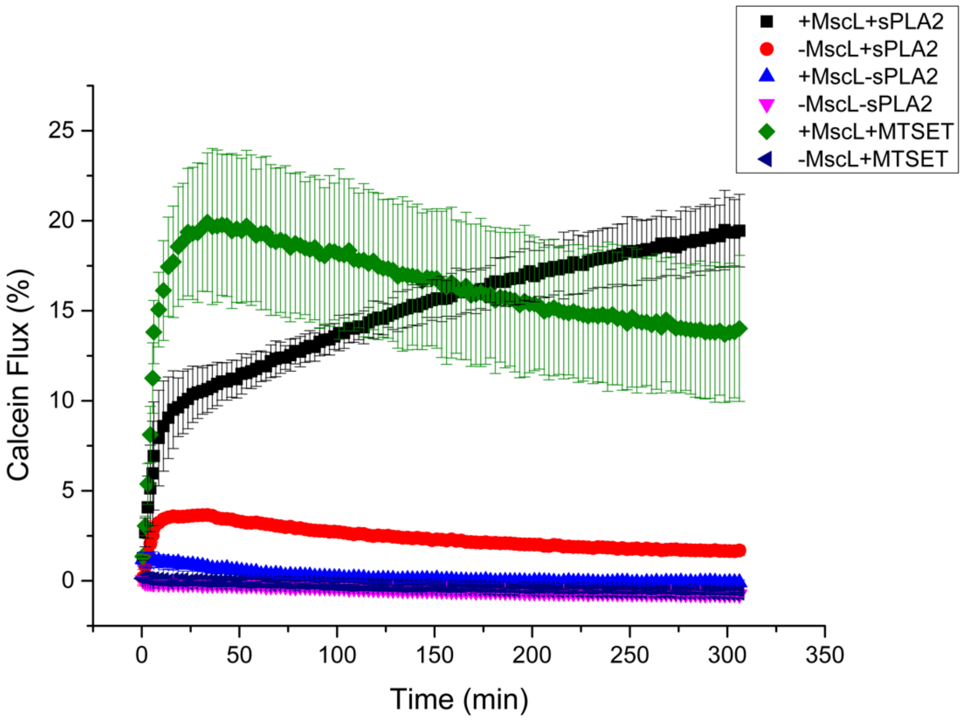

Figure S5. Vesicles containing reconstituted MscL respond to enzymatic and chemical activators of the channel. Purified MscL is necessary for vesicles to respond to enzymatic (bee venom secretory phospholipase A2, 3 U/ml) or chemical ([2-(Trimethylammonium)ethyl]methanethiosulfonate, 1 mM) activation, as demonstrated by significant calcein flux occurring over 5 hours for these conditions. If the channel is removed, calcein flux is either significantly reduced or abolished completely when using enzymatic or chemical activation respectively. Error bars = 1 S,D., n =3.

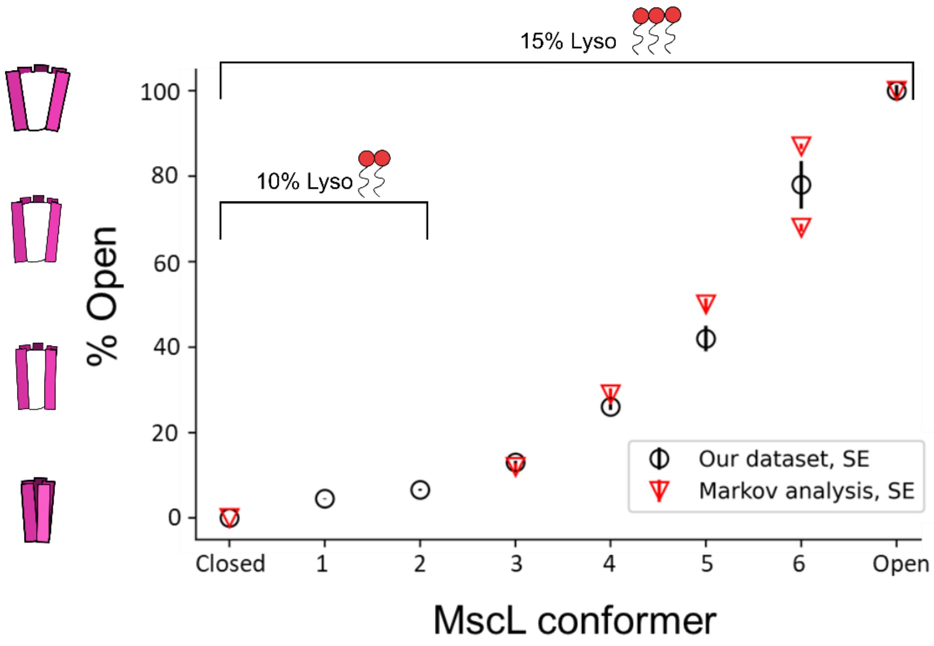

Figure S6 – Graphical summary of analysed events for MscLG22C F93W gating in the presence of LysoPC compared to reference dataset.[9]

∆ pA	nS	n	SE (pA)	SE (%)	% of open state (measured)	% of open state (reference)
14.6	0.15	27	0.32	2.15	4.5	n/a
21.2	0.21	5	0.78	3.69	6.6	7[8]
42.3	0.42	3	1.37	3.23	13	12[9]
84.3	0.84	3	1.80	2.13	26	29[9]
135	1.35	3	7.80	5.79	42	50[9]
252	2.52	3	14.73	5.84	78	68 / 87[9]
323 (Open)	3.23	3	3.66	1.14	100	100[9]

Table S1 – Summary of analysed events for MscLG22C F93W gating in the presence of LysoPC. Table and Figure S6 illustrate the results from histogram analysis of the total data set, SEs observe no overlap between populations. Strong agreement is observed when compared to literature characterisation of MscL sub conducting states. State 6 (2.52 nS / 78%) is likely to contain analyses from states 68 / 87 from the literature dataset, due to lack of events from 2.2 – 2.8 nS, the data has been presented as a mean. State 2 has been observed previously[1] but has not been included in the Markov model[2] and hence is not included as a reference data point in Figure S6.

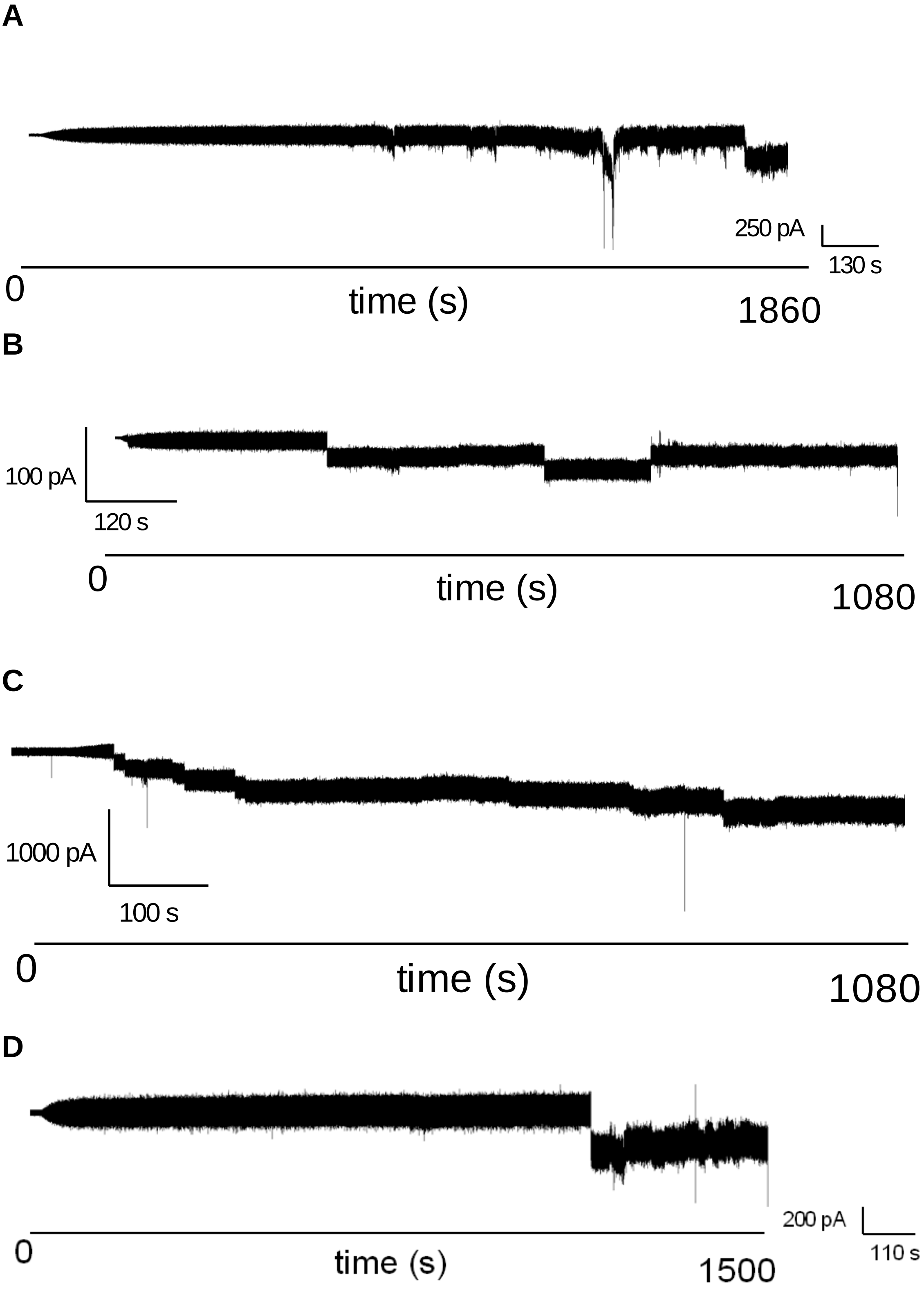

Figure S7. Unfiltered traces for MscL DIBs containing 15% LPC.

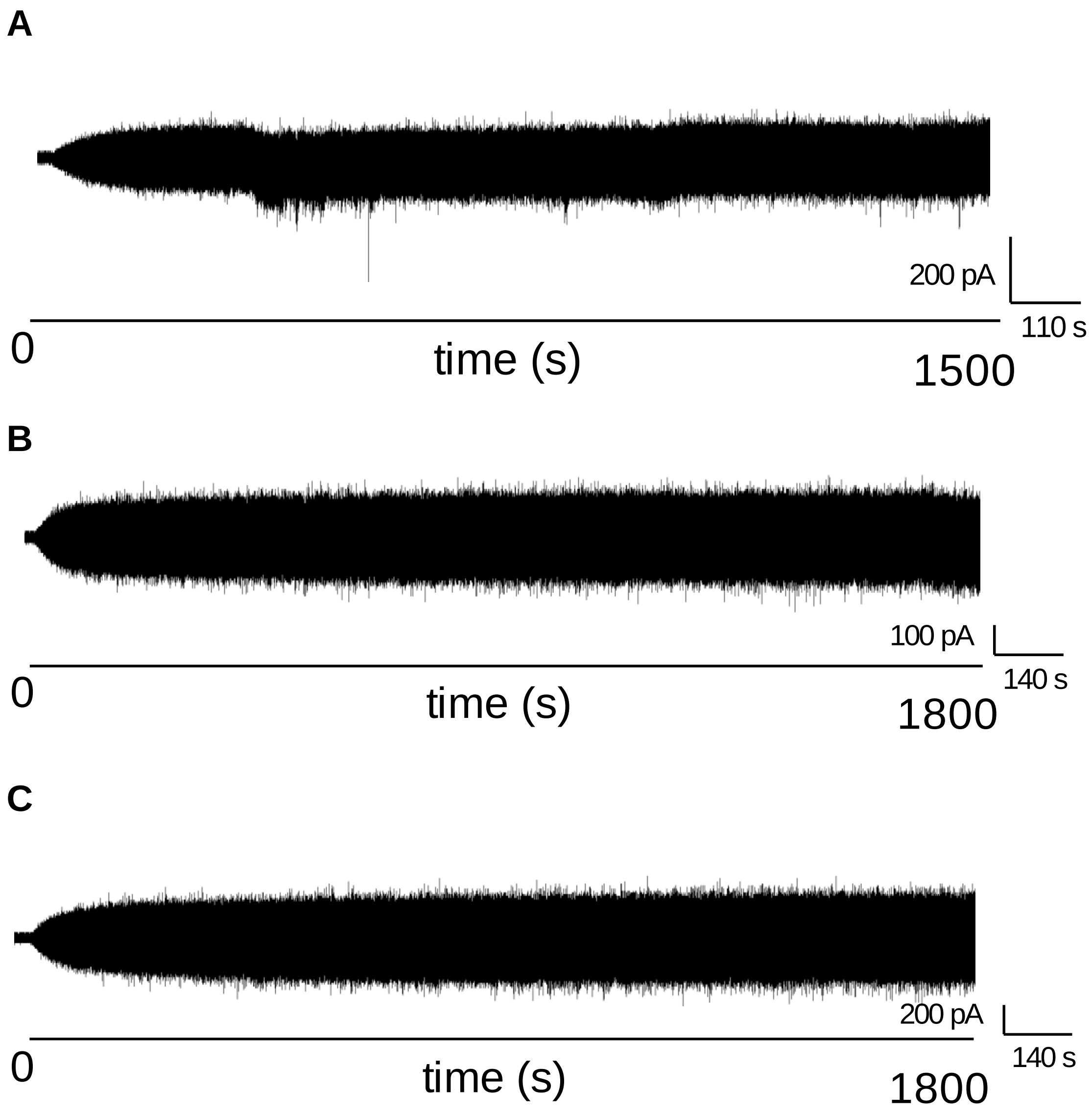

Figure S8. Unfiltered traces for MscL DIBs containing 10% LPC.

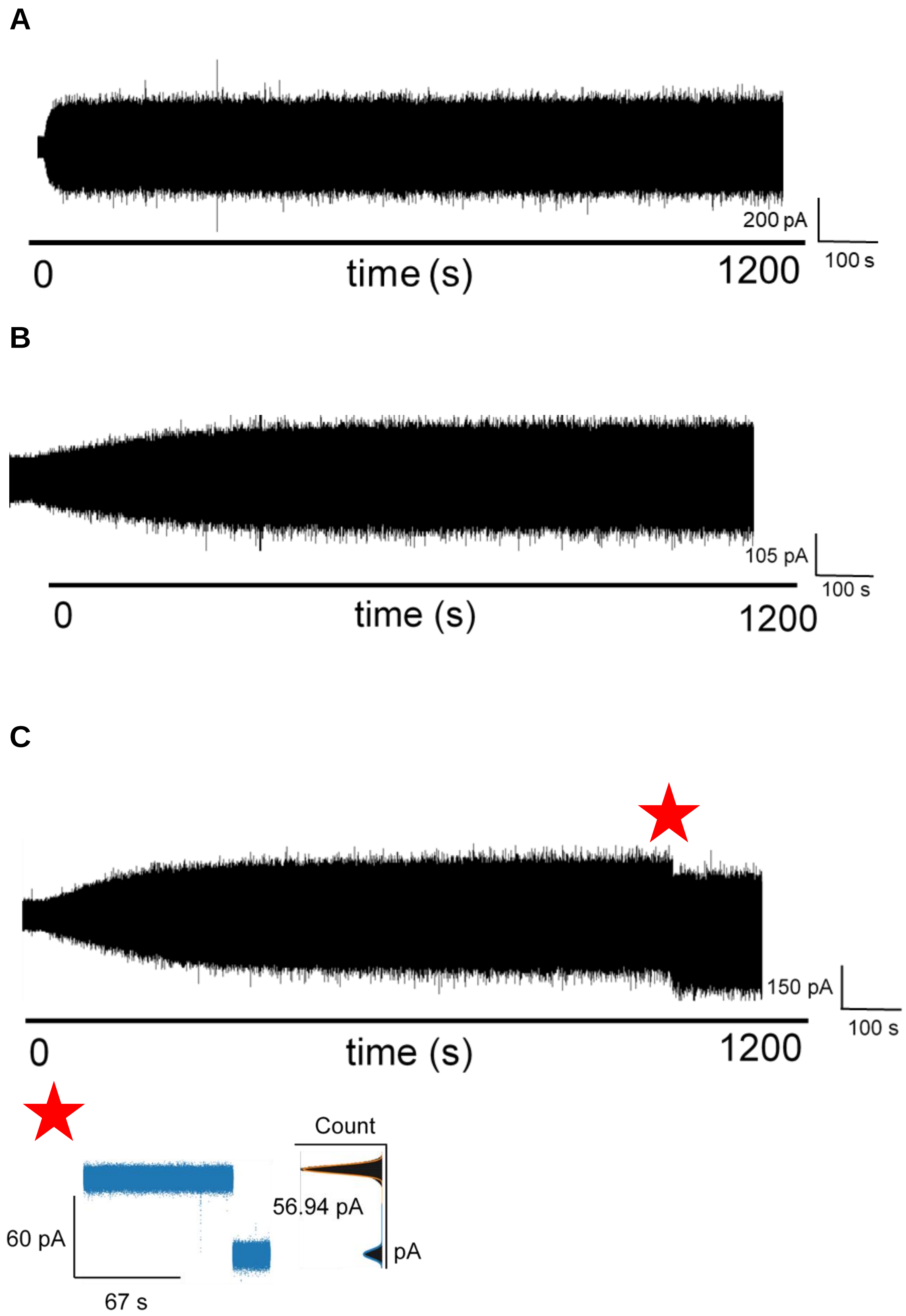

Figure S9. Unfiltered traces for MscL DIBs containing 5% LPC. One sustained opening event observed at 56.94 pA.

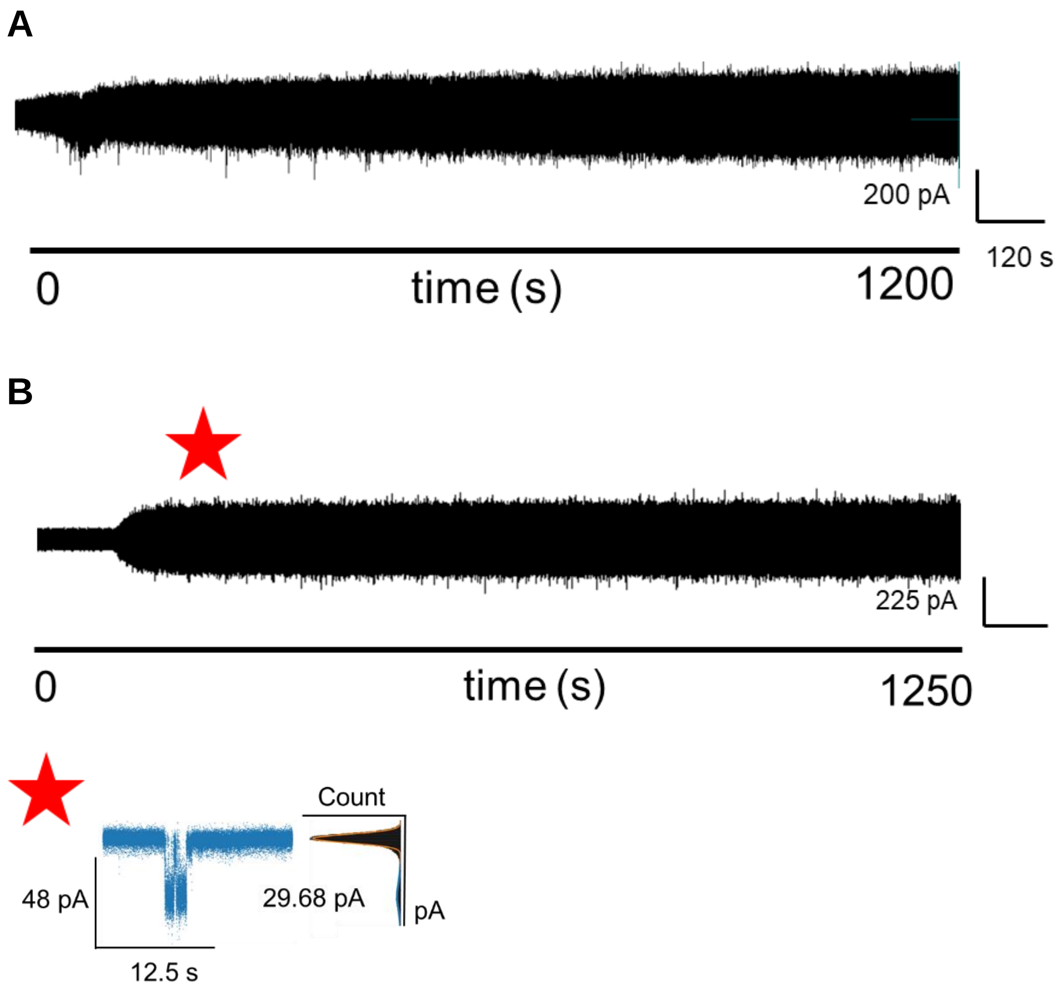

Figure S10. Unfiltered traces for MscL DIBs containing 0% LPC. Negligible events detected in symmetric DIBs, flickering opening event in B, t_0 = 217 s – Value indicative of the flickering of possibly two channels at the 15 pA level, or an otherwise unidentified sub conductive state, open for approximately 2 s.

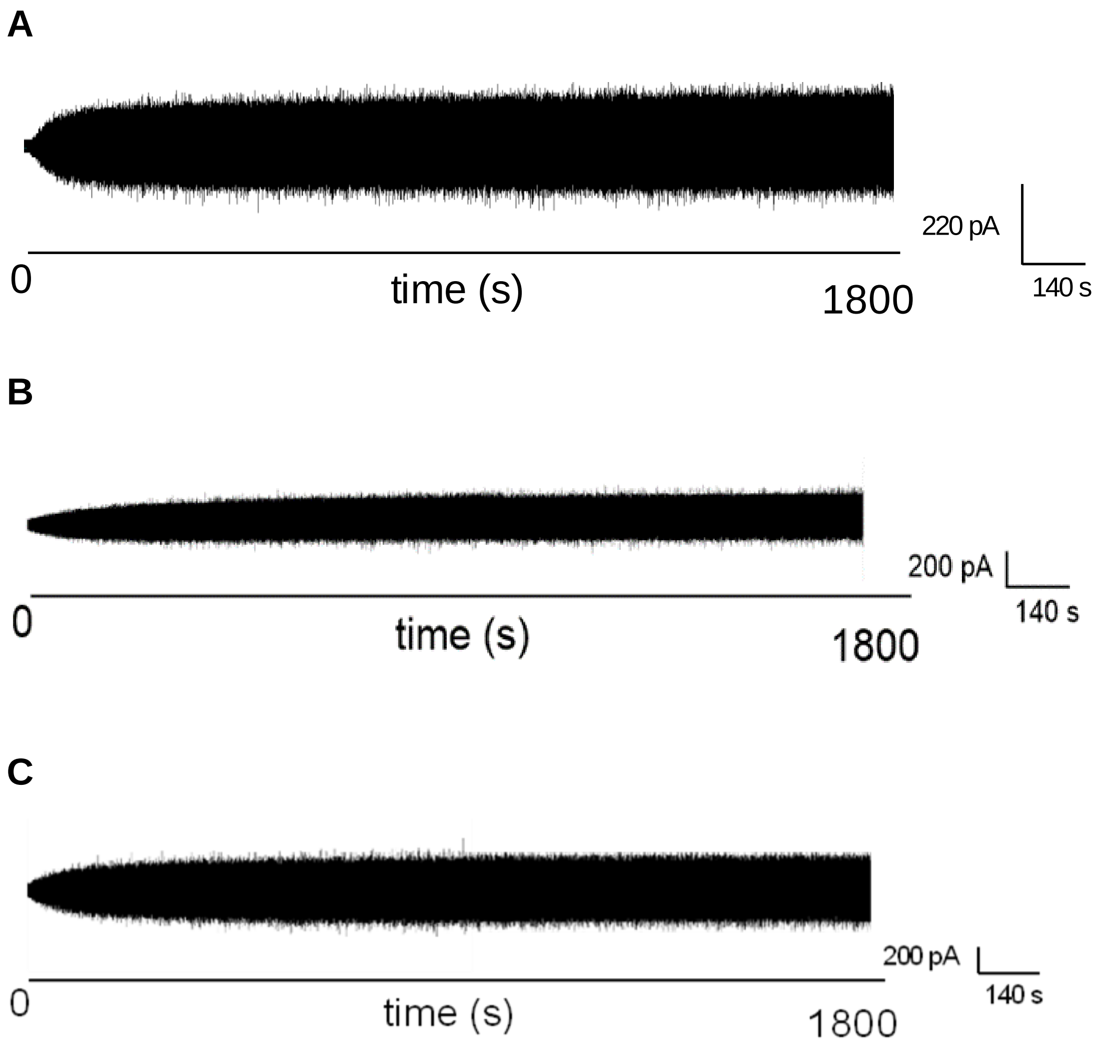

Figure S11. Unfiltered traces for DIBs containing 15% LPC without MscL (asymmetric). No opening events found in any trace.

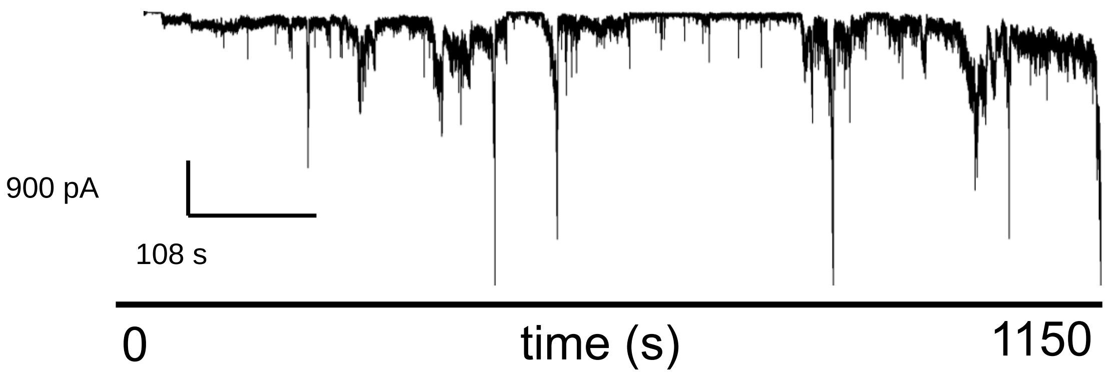

Figure S12. Unfiltered trace for symmetric DIBs containing 15% LPC in each leaflet (symmetric DIB).

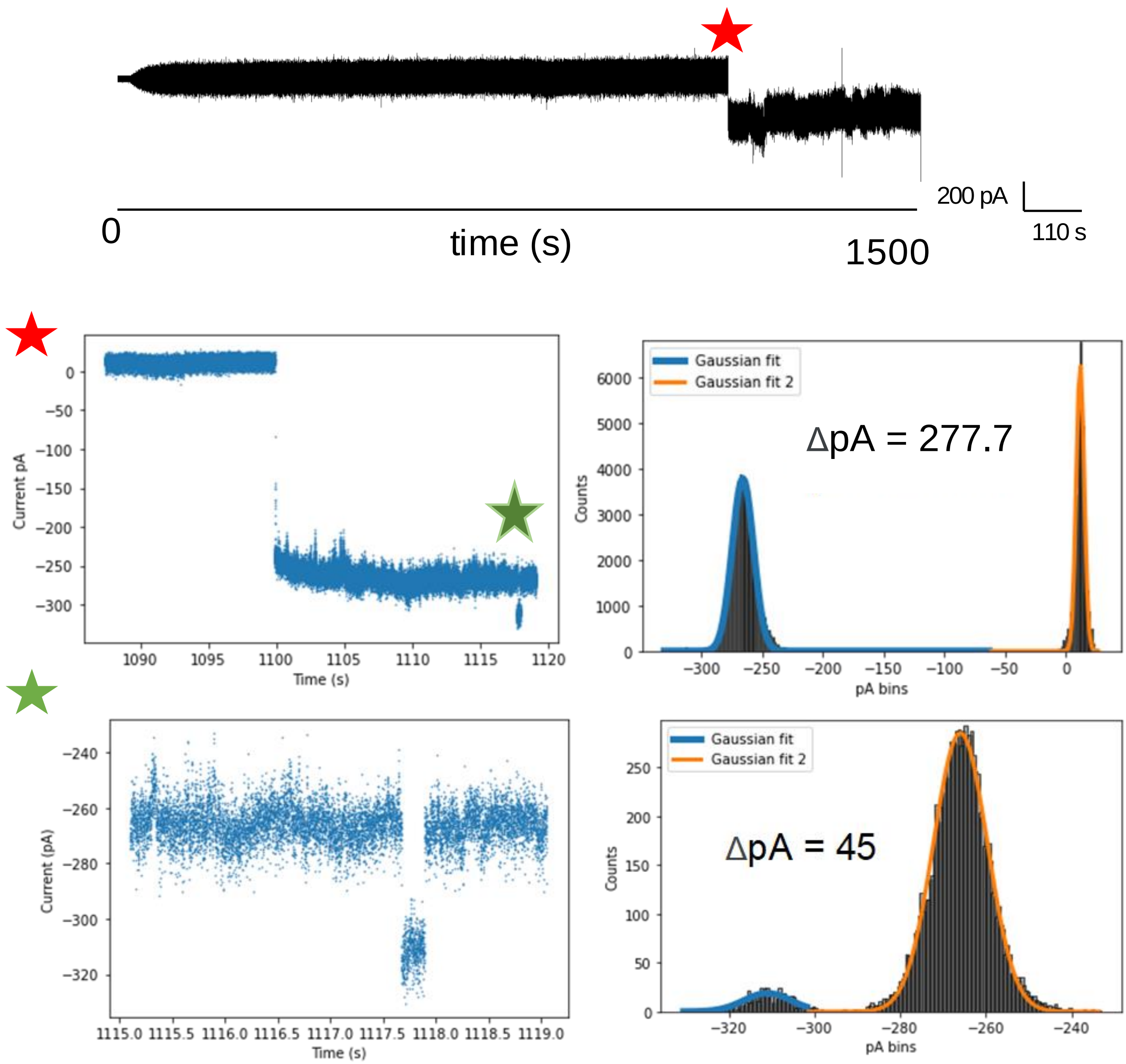

Figure S13 – Analysis of a full opening event - The following shows the sustained opening of a channel at around ~280 pA (red star) before further briefly opening to what appears a full opening of ~323 pA (green star). Note, this fully opened state appears to be sustained for around 0.3 s before closing back to the ~ 280 level.

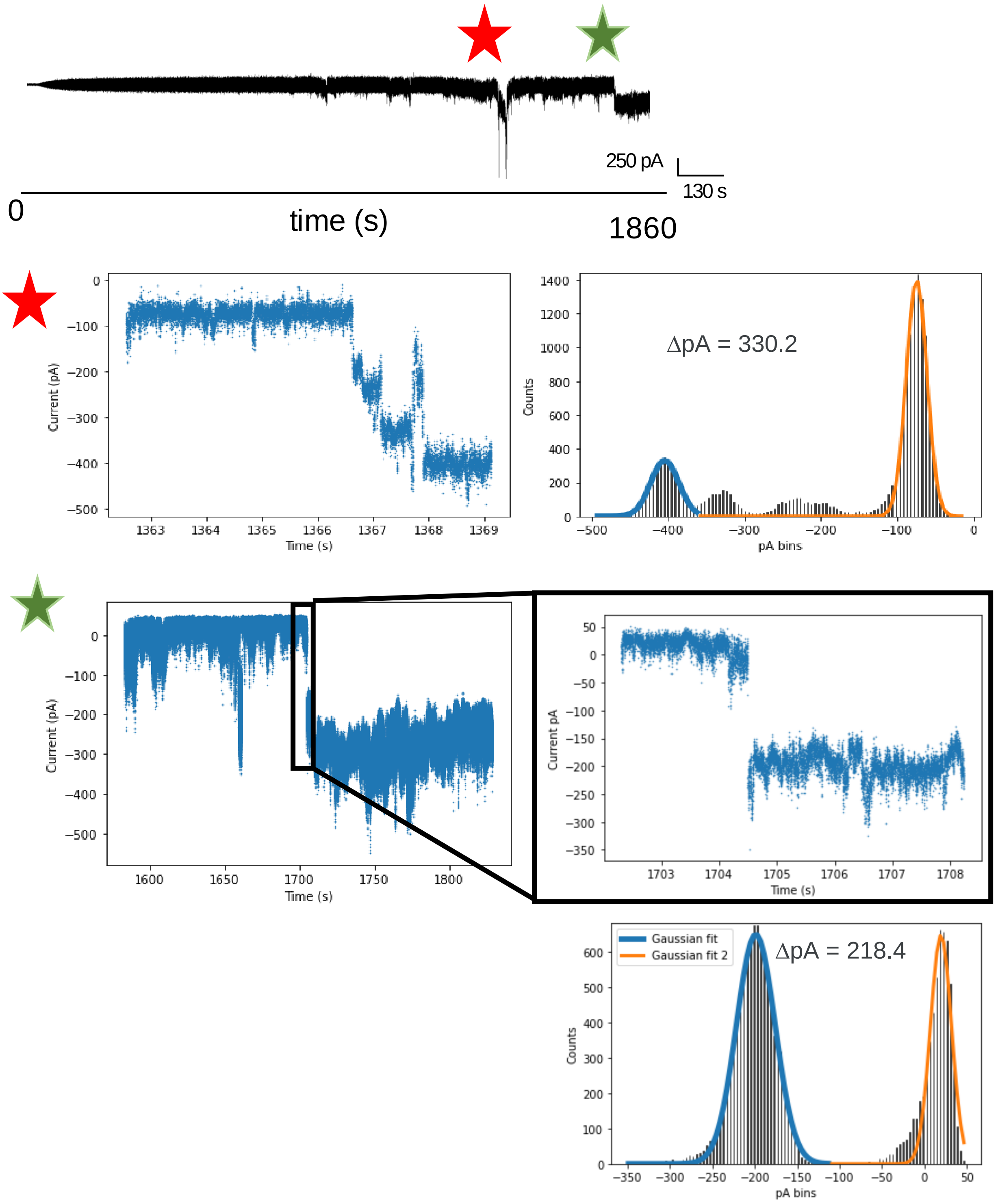

Figure S14: Illustrating large opening events going via sub conductive states:

Red star: Trace shows what appears a full opening event passing via distinct sub conductive states, note that sub opening events had occurred prior to this event, hence deviation from baseline. The difference in amplitude shown in the histogram approach, between the top (orange) and bottom (blue) states is 330.21 pA. To note, the penultimate level, which is resolved as the distribution to the right of the fitted blue distribution, is Δ258.23 pA. A rough approximate for the other two initial opening states is ~120, 160 pA.

Green Star : Zoom in of sustained opening event, shows a clear opening of a sub state, where histogram analysis indicates a Δ218.45 pA, to note, we attribute the further drop to a full opening event at 314.67 due to this being sustained in the trace however the increase in noise present here makes quantitative assessment without further data processing challenging.

A

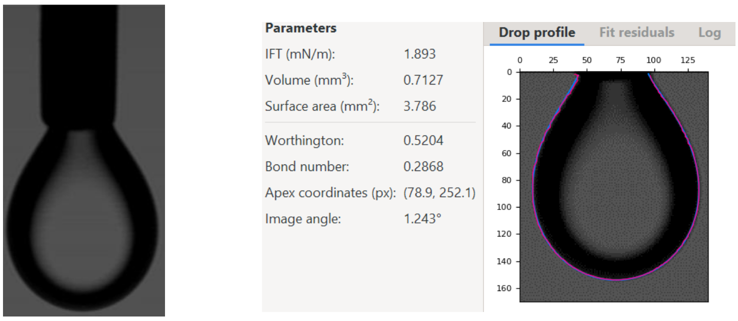

B

Composition (DOPC:DOPG:LPC – (n))	IFT (mN/m)	SD (mN/m)	Average volume (mm³)	Average Worthington (Wo)
95:5:0 (6)– 0% LPC	1.83	0.229	0.370	0.282
90:5:5 (4) – 5% LPC	1.56	0.314	0.338	0.515
85:5:10 (6) – 10% LPC	1.20	0.514	0.567	0.427
80:5:15 (8) – 15% LPC	1.09	0.705	0.170	0.292

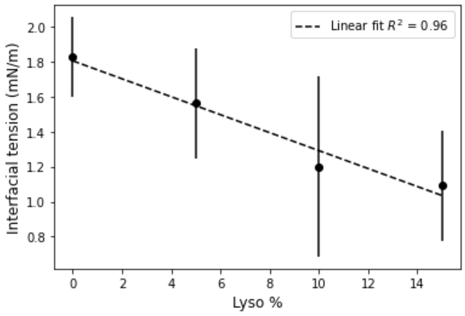

Figure S15. DSA uncovers a linear relation between LPC content and monolayer IFT. A. Example of DSA, with the output from OpenDrop software included, here shown for our 95:5 DOPC : DOPG composition (0% LPC). **B.** Table showing a summary of IFT measurements using DSA with varying LPC molar %, all compositions are representative of droplets used in electrophysiology experiments. Error bars = 1 S.D. Plot indicates a linear relationship between monolayer surface tension and molar% LPC (R^2 = 0.96).

Note S1. Python pipeline to determine ∆pA for protein openings.

Analysis of electrophysiology data is known for its non-triviality and our analysis was improved by the development of a python pipeline. A standardised approach for this problem utilises Clampfit software from Molecular Devices, we sought to use this method to develop our pipeline. A typical result from a Clampfit approach is as follows:

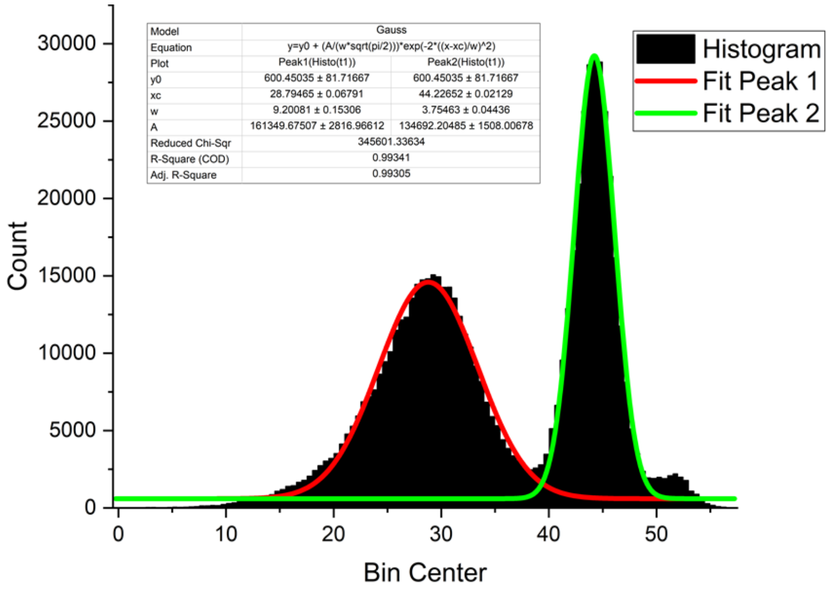

Figure S16. Example of Gaussian fit method via OriginPro Data is selected in Clampfit and ran through a low pass filter, and a histogram produced before export to OriginPr0 for fitting with a Gaussian function and plotting. The difference in amplitudes is then determined, here the ∆pA = 15.44 and the value for this same region when determined via our python method (as presented in in figure 3) = 15.09, t_0 516 s, Figure 3 B, 10% LysoPC gating.

Our pipeline was designed around a typical analysis with Clampfit. The data was first loaded in using the pyabf library (Harden, SW (2020). pyABF 2.2.3. Available: https://pypi.org/project/pyabf/). A 50kHz, 2000 s trace is typically ~ 2 GB and to enhance processing, the data is reduced from 50 kHz to 2.5 kHz. The user then finds a section of the trace and utilises a function which performs a histogram (numpy) and Gaussian fitting (scipy.optimize) of this section, the difference in amplitude is then determined. To ensure that our data handling procedures had no impact on our conclusions, here we compared the same regions of trace analysed via both programmes.

**States (py)**	14.5 ± 0.26	21.3 ± 0.54
**States (Clampfit)**	14.9 ± 0.28	21.9 ± 0.65

Table S2. Summary of pA states observed with a histogram and Gaussian fit approach across both 10 and 15 % LysoPC activated small MscL gatings with two different analytical programmes Mean values presented with SE. For Clampfit, n = 8 and 4 for the observed ~14.8, 21.9 states respectively, presented further in Table S3.

Table S2 shows that any discrepancy between the analytical processes is shown to be negligible. A slight increase of the openings is seen with the Clampfit approach, possibly due to a slightly larger bin size in the Clampfit. We found that the python pipeline allowed the analyst to improve the speed of their work whilst reducing human error in the analytical process. It is worth acknowledging that variance in bin size and width within the analytical approach is likely to drive variance in ΔpA, not readily addressed in the current literature. In our pipeline, this was easily optimisable for each trace. Alternatively, this discrepancy may be due to signal reduction in python from 50 kHz to 2.5 kHz. Finally, a presentation of a range of different openings affirms the minimal discrepancy between analytical approaches (Table S3 below).

Lyso%	Repeat	Length	ΔpA (py)	ΔpA (Clampfit)
15	1	Sustained	23.24	23.75
		Sustained	21.74	21.91
		Sustained	21.75	21.72
15	2	Sustained	131.4	136.3
		Sustained	81.14	80.33
		Sustained	83.12	82.31
		Sustained	88.5	94.46
		Sustained	152.7	149.2
		Shorter	14.5	14.63
		Shorter	15.27	15.76
		Shorter	16.03	13.61
		Shorter / Sustained	13.11	13.72
		Shorter / Sustained	14.55	14.84
		Shorter	12.34	14.37
15	3	Shorter	34.71	35.9
		Shorter	54.21	54.41
10	1	Sustained	15.83	16.58
		Shorter	20.47	20.61
		Shorter	15.87	14.48
10	2	Shorter	15.09	15.44

Table S3. Comparative data from analytical methods. All data, including larger state changes illustrates negligible variance in ΔpA across both methods. The above data set was averaged in Table S2. A range of sub conductive states were sampled.

Author Contributions

RS and JWH designed and conducted experiments, wrote the manuscript and performed data analysis, MF designed experiments, performed data analysis and helped write the manuscript, JG conducted experiments and performed data analysis, OC, RL, JDH and PJB designed experiments and helped to revise the manuscript.

References

1. J. W. Hindley, D. G. Zheleva, Y. Elani, K. Charalambous, L. M. C. Barter, P. J. Booth, C. L. Bevan, R. V Law and O. Ces, *Proc. Natl. Acad. Sci. U. S. A.*, 2019, **116**, 16711–16716.
2. A. M. Powl, J. M. East and A. G. Lee, *Biochemistry*, 2008, **47**, 4317–4328.
3. K. Charalambous, P. J. Booth, R. Woscholski, J. M. Seddon, R. H. Templer, R. V Law, L. M. C. Barter and O. Ces, *J. Am. Chem. Soc.*, 2012, **134**, 5746–5749.
4. S. Haylock, M. S. Friddin, J. W. Hindley, E. Rodriguez, K. Charalambous, P. J. Booth, L. M. C. Barter and O. Ces, *Commun. Chem.*, 2020, Just Accepted.
5. N. E. Barlow, H. Kusumaatmaja, A. Salehi-Reyhani, N. Brooks, L. M. C. Barter, A. J. Flemming and O. Ces, *J. R. Soc. Interface*, 2018, **15**, 20180610.
6. G. A. Venkatesan, J. Lee, A. B. Farimani, M. Heiranian, C. P. Collier, N. R. Aluru and S. A. Sarles, *Langmuir*, 2015, **31**, 12883–12893.
7. J. D. Berry, M. J. Neeson, R. R. Dagastine, D. Y. C. Chan and R. F. Tabor, *J. Colloid Interface Sci.*, 2015, 454, 226–237.
8. C. S. Chiang, A. Anishkin and S. Sukharev, *Biophys. J.*, 2004, **86**, 2846–2861.
9. I. M. Almanjahie, R. N. Khan, R. K. Milne, T. Nomura and B. Martinac, *Eur. Biophys. J.*, 2015, **44**, 545–556.
10. H. Kaluarachchi, J. F. Siebel, S. Kaluarachchi-Duffy, S. Krecisz, D. E. K. Sutherland, M. J. Stillman and D. B. Zamble, *Biochemistry*, 2011, **50**, 10666–10677.
